# A novel low-complexity post-processing algorithm for precise QRS localization

**DOI:** 10.1186/2193-1801-3-376

**Published:** 2014-07-25

**Authors:** Pedro Fonseca, Ronald M Aarts, Jérôme Foussier, Xi Long

**Affiliations:** Department of Electrical Engineering, Eindhoven, University of Technology, Postbus 513, 5600 MB Eindhoven, The Netherlands; Philips Research, High Tech Campus 34, 5656 AE Eindhoven, The Netherlands; Philips Chair for Medical Information Technology, RWTH Aachen University, Pauwelsstraße 20, D-52074 Aachen, Germany

**Keywords:** Electrocardiography, QRS localization, Trigger jitter, Motion artifacts

## Abstract

Precise localization of QRS complexes is an essential step in the analysis of small transient changes in instant heart rate and before signal averaging in QRS morphological analysis. Most localization algorithms reported in literature are either not robust to artifacts, depend on the sampling rate of the ECG recordings or are too computationally expensive for real-time applications, especially in low-power embedded devices. This paper proposes a localization algorithm based on the intersection of tangents fitted to the slopes of R waves detected by any QRS detector. Despite having a lower complexity, this algorithm achieves comparable trigger jitter to more complex localization methods without requiring the data to first be upsampled. It also achieves high localization precision regardless of which QRS detector is used as input. It is robust to clipping artifacts and to noise, achieving an average localization error below 2 ms and a trigger jitter below 1 ms on recordings where no additional artifacts were added, and below 8 ms for recordings where the signal was severely degraded. Finally, it increases the accuracy of template-based false positive rejection, allowing nearly all mock false positives added to a set of QRS detections to be removed at the cost of a very small decrease in sensitivity. The localization algorithm proposed is particularly well-suited for implementation in embedded, low-power devices for real-time applications.

## Introduction

Automatic QRS detection is a basic and widely used technique in the analysis of electrocardiographic (ECG) recordings and a significant amount of research effort has gone into developing automatic algorithms with high sensitivity and positive predictive value (PPV) (Köhler et al. 2002). However, despite being able to correctly detect the presence of QRS complexes, most algorithms fail to provide their precise location. In fact, standards for evaluating the performance of QRS detectors are often not strict enough. For example the ANSI/AAMI EC57 norm (EC57:^1998^/(R)2008 AS (1998)) recommends a margin of 150 ms when evaluating QRS detections. In a recording where the subject has an average heart rate of 60 beats per minute (bpm), this margin corresponds to 15% of the average length of a beat. This criterion is clearly meant as a guideline for evaluating the detection performance. Although it may suffice in applications where the correct detection of QRS complexes is more important than their localization, there are many areas in which the detection of small transient changes in heart rate is crucial.

For example, in the field of sleep research, Catcheside et al. (Catcheside et al. 2001) reported how the heart rate increases by approximately 14% within two seconds of the onset of arousal-inducing tones during sleep. This was further confirmed by Bangash et al. (Bangash et al. 2008) who observed an increase of 10% (during REM sleep) and 15% (during non-REM) three seconds after an arousal-inducing tone, followed by a decrease of 15% (REM) and 25% (NREM) five seconds after the tone onset.

Another example comes from the area of QRS morphology analysis in high-resolution ECG. If the localization of QRS complexes before signal averaging^a^ is not precise enough, the resulting signal will suffer from a low-pass filtering effect which may hide relevant high-frequency low-level potentials (Breithardt et al. 1991; Jané et al. 1991). Since these potentials can help, for instance, in the analysis of ventricular late potentials in patients recovering from myocardial infarction, guidelines have been proposed describing the desired precision of QRS localization in terms of trigger jitter (standard deviation of the localization error), which should be below 1 ms (Breithardt et al. 1991).

A third area in which the precise localization of QRS complexes is of paramount importance is in heart rate variability (HRV) analysis. HRV analysis is a widely used technique to assess different aspects of the autonomic nervous system (ANS) and has been shown to be clinically relevant for many different applications, such as a predictor of cardiovascular disease and mortality (Thayer et al. 2010), to assess possible ANS dysfunctions in patients with chronic obstructive pulmonary disease (Volterrani 1994), and for screening of sleep disorders and evaluation of sleep quality (Stein and Pu 2012). HRV time series are usually computed from the intervals between consecutive QRS complexes and it is recommended that the ECG is sampled at high enough sampling rates (250-500 Hz or higher) to guarantee a small trigger jitter in the localization of the R wave fiducial point, needed for the accurate estimation of HRV parameters (Task Force of the European Society of Cardiology and the North American Society of Pacing and Electrophysiology 1996).

Several localization methods have been proposed to address the issue of precise QRS localization. However, most either depend on the sampling rate of the original ECG (such as cross-correlation- and normalized integrals-based methods (Jané et al. 1991)), are too sensitive to noise or to signal clipping (such as threshold-based methods (Jané et al. 1991)) or are computationally complex and not adequate to real-time applications (such as interpolation and curve fitting (Bragge et al. 2005)). Other techniques such as vectorcardiographic loop alignment, depend on the recording of multiple ECG leads (Sörnmo 1998). The increase in the computational power of modern computers allows techniques such as cross-correlation localization to overcome limitations in regard to the sampling rate by simply upsampling the ECG signal. However, this step renders these algorithms unsuitable for low-power, embedded processors for wearable or even implantable devices. Furthermore, template-based localization algorithms such as cross-correlation are not adequate for real-time processing since they require a template to first be built from QRS complexes before localization can finally take place.

The objective of this paper is to propose and evaluate a post-processing method for precise localization of QRS complexes in single-lead ECG recordings. The proposed method, henceforth referred to as the *slope* algorithm, should address the following requirements:

- **Complexity** - the algorithm should have low complexity, *O*(*n*), to enable real-time processing with modest hardware requirements and integration in low-power embedded devices.
- **Trigger jitter** - the algorithm should yield at least the same trigger jitter as the more computationally complex cross-correlation-based method in high resolution ECG, and better trigger jitter at lower sampling frequencies, to enable a high localization precision in ECG recorded with lower sampling frequencies.
- **Localization error** - the algorithm should have low average localization error, below 10 ms (1% of the average length of a beat for a recording where the average heart rate is 60 bpm), to enable applications (such as sleep arousal detection) which depend on the detection of small transient changes in interbeat interval length.
- **Robustness** - the algorithm should be robust to the presence of noise and movement artifacts, to enable its application in recordings performed in uncontrolled (non-laboratory) conditions; in addition, the algorithm should handle signal clipping, a common occurrence in recordings during sleep (Redmond and Heneghan 2006).
- **Agnostic** - the algorithm should be agnostic to the QRS detection algorithm used, and perform equally well regardless of the method.

Additionally, it will be shown how template matching can be used to reduce the number of false positive detections and that the localization step performed by this post-processing algorithm is essential for that purpose. Template matching can be used, for instance, to exclude false positive detections which lead to erroneously short estimates of interbeat intervals, or to exclude beats with aberrant QRS morphology before averaged complexes are analyzed (Sörnmo 1998).

## Methods

One of the main problems of most QRS detectors is that in the presence of noise, movement artifacts or signal clipping, they do not provide precise estimates of the location of the peaks. Consequently, a post-processing localization step is usually performed to obtain a more precise location of detected complexes (Köhler et al. 2002).

### QRS Detection

The localization algorithm described in this paper can be used after any of the vast number of QRS detectors reported in literature (Ferreira et al. 2013; Friesen et al. 1990; Köhler et al. 2002). For the purpose of evaluating its performance, three popular detectors described in literature were used: the Hamilton-Tompkins (HT) detector (Hamilton 2002; Hamilton and Tompkins 1986), an envelope-based detector by Nygårds and Sörnmo (NS) (Nygårds and Sörnmo 1983) and a filter bank-based detector by Afonso, Tompkins et al. (AT) (Afonso et al. 1999).

### Filtering

Before the signal is processed, baseline wander is first removed with a linear phase high-pass filter using a Kaiser window of 1.016 sec, with a cut-off frequency of 0.8 Hz and a side-lobe attenuation of 30 dB (van Alsté et al. 1986). The coefficients of the impulse response were determined by computer-aided filter design with the software Matlab R2012b (The MathWorks Inc., Massachusetts).

### Localization

**Figure 1:**
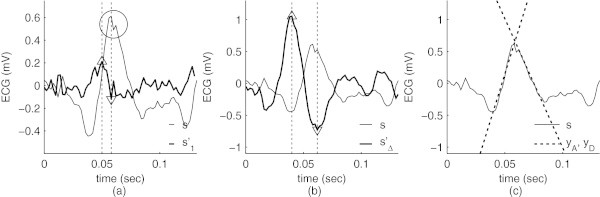
**Estimating the location of the R wave peak for a QRS complex with an artifact: (a) signal** ***s*** **with a motion artifact (indicated with a circle) and differentiated signal with a step size of 1 sample,** ***s’***_***1***_ **(in bold) with maximum and minimum derivative values indicated by up- and down-pointing triangles, respectively; (b) differentiated signal with a step size Δ of 10 samples,** ***s’***_**Δ**_ **(in bold); (c) tangents to the ascending and descending slopes of the QRS complex,** ***y***_***A***_ **and** ***y***_***D***_ **, with the peak localized by their intersection.**

The level of noise, the amount of artifacts and the possible presence of signal clipping, common in recordings during sleep, all have a significant impact in the localization performance of most QRS detectors. Consider the example of a QRS complex with a motion artifact which distorts the R peak, as illustrated in Figure 1a (highlighted with a circle). Although most QRS detectors will correctly identify the presence of this complex, they will usually yield an incorrect location, usually the local maximum.

This common type of problems is first addressed by observing that the Q-to-R and R-to-S amplitudes of a QRS complex are normally much larger than the amplitude of measurement noise. They are usually also larger than the amplitude of artifacts which do not distort the shape of the complex beyond the point that the R wave is no longer distinguishable (Friesen et al. 1990). Note that this might not hold in the presence of intense body movements. However, it is arguable whether the QRS detectors used before post-processing would be able to detect the presence of such peaks anyway.

Because the R waves are very steep, signal differentiation usually gives the ascending and descending slopes as local maxima and minima, respectively. This simple approach has been widely used (Köhler et al. 2002), and remains the basis of some of the most popular algorithms to date (Hamilton and Tompkins 1986; Pan and Tompkins 1985). However, it suffers from some drawbacks, notably in the presence of artifacts which cause sudden, drastic changes in the signal amplitude. In such cases it often occurs that the local maxima of the differentiated signal for these artifacts is larger than the maxima which correspond to the actual slopes of the R wave (Figure 1a). This problem can be solved simply by observing that QRS complexes have a predictable length: in the absence of a medical (cardiac) condition, they last in average about 0.08 seconds (Rangayyan 2001). Instead of differentiating the ECG signal on a sample-to-sample basis, a larger step can be used to more accurately calculate the slope of the tangent to the signal (Figure 1b). Local maxima and minima will indicate the beginning of the slopes which are simultaneously steep and have at least a given duration. The intersection of the tangents to these slopes yields the location of the R wave peak (Figure 1c). The *slope* algorithm is formally described as follows:

1. Differentiate the filtered ECG signal with a step size Δ to obtain *s'*1
2. For each base peak location *p*_*i*_ estimated by a QRS detector:
  - Find the location of the local maximum  in a window centered around *p*_*i*_ , 2
    with 3
    where *W* corresponds to the expected length (in samples) of the QRS complex (0.08 sec).
  - Find the location of the local maximum and the local minimum of *s* ' in a window respectively before and after 45
  - Compute the slope and the y-intercept of the tangent to the ascending slope, *y*_*A*_6
    with 78
    and to the descending slope, *y*_*D*_9
    with 1011
  - Compute the location of the intersection *n*_*I*_ between the two tangents, 1213

Note that the tangent intersection *n*_*I*_ does not necessarily correspond to the exact (integer) location of a sample in the discretized ECG. This means that the location of the peak can be computed with sub-sample precision and has as an important consequence, as will be shown, that the localization error is to a certain extent independent of the sampling rate of the ECG signal.

The differentiation step Δ is an important parameter in the algorithm. As explained in Appendix A, this factor should be as large as possible to minimize the localization error. However, it should not be larger than the length of the slope. In order to increase the robustness of the algorithm to clipping artifacts which cause the slopes to be shortened not only in amplitude but also in duration, a step size corresponding to approximately half of the expected length of each slope was chosen. Considering that each QRS complex lasts about 80 ms, and that each slope lasts around 40 ms, a step size of 20 ms was chosen and experimentally found to be adequate. Note that when analyzing unclipped signals this parameter can be increased in order to further reduce the trigger jitter, as explained in Appendix A; lowering the parameter increases the trigger jitter in the presence of noise. Figure 1a illustrates (in bold) the signal obtained after single-sample differentiation. The artifact at the center of the R wave introduces additional local minima which do not correspond to the descending slope of the QRS complex. Figure 1b illustrates the signal obtained after differentiating the ECG with a Δ factor of 10 samples (20 ms at a sampling frequency of 500 Hz). The maximum and minimum values now correspond to the beginning of the steepest parts of the slopes. After identifying the parameters of the tangent to the ascending and descending slopes, their intersection yields the location of the R wave peak (Figure 1c).

As explained in Appendix B, this algorithm has a complexity of *O*(*n*), lower than that of cross-correlation-based methods.

### Template matching

Template matching can be used to reduce the number of false positive detections in a recording. The template should be chosen such that it represents the morphology of the QRS complexes in an ECG recording and can be obtained by averaging the signal around the location of each peak in a window with a length equal to the median duration of the beats in each recording.

**Figure 2:**
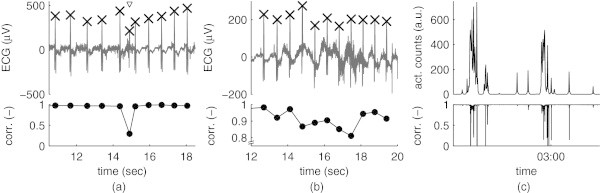
**Template matching after QRS detection (with the Hamilton-Tompkins detector): (a) in the presence of a false positive (indicated with a downward arrow); (b) in the presence of complexes heavily distorted by noise; (c) compared with actigraphy for a full night recording.**

After the template is estimated, the correlation between each detected complex and the template can be computed. Under the assumption of monomorphic QRS complexes, false positive locations should have a lower correlation value (Figure 2a) than undistorted complexes. Complexes slightly distorted by noise should also yield a lower correlation value, but as long as they retain some of their characteristics the correlation should still be higher than of false positives (Figure 2b). As an example of the impact of body movement artifacts, Figure 2c illustrates the correlation obtained with complexes detected and localized in an ECG recorded simultaneously with actigraphy (Actiwatch Spectrum, Philips Electronics) during a full night. As it can be easily seen, the correlation is inversely proportional to the amplitude of the actigraphy peaks.

### Evaluation

#### Data sets

The *slope* algorithm was tested in two publicly available data sets. The first comprises 18 long-term ECG recordings (lead II) of subjects with no significant arrhythmias from the MIT-BIH Normal Sinus Rhythm Database (MIT-BIH) (Goldberger et al. 2000; Moody and Mark 2001). Each recording has a sampling rate of 128 Hz and an average length of 24.3 ± 0.83 hour. This data set was chosen since it comprises full day recordings, including periods with intense motion artifacts, naturally occurring in this type of monitoring scenarios.

The second data set comprises 79 high-resolution ECG recordings (lead II) of subjects annotated as healthy controls in the PTB Diagnostic ECG Database (PTBD) (Bousseljot et al. 1995; Goldberger et al. 2000). Each recording has a sampling rate of 1000 Hz and an average length of 118.9 ± 3.4 sec.

#### Cross-correlation localization

As discussed, localization has been traditionally performed for the purpose of signal averaging in the area of QRS morphology analysis where it is crucial to have a low trigger jitter. One of the most successful methods uses cross-correlation between a template (built by averaging QRS complexes detected with a QRS detector) and each complex to improve the estimated location. Since this is one of the most successful localization algorithms reported in literature, especially when used on high-resolution (or upsampled) ECG signals, it will be used to establish the ground-truth locations of QRS complexes in our database and as reference to evaluate the *slope* algorithm.

An implementation of this method, henceforth referred to as the *xcorr* algorithm, is described as follows:

- Remove baseline wandering using the filtering procedure described in an earlier section.
- Compute a template of the QRS complexes based on the initial locations, using the template algorithm described in a later section.
- For each complex, find the location which maximizes the cross-correlation between the template and the ECG signal around the original location.
- Build a new template based on the adjusted locations.
- For each complex, store as the final location of its R wave peak the location that maximizes the cross-correlation between the new template and the ECG signal around the adjusted location.

#### Ground-truth

The MIT-BIH data set includes annotated QRS locations per recording. Unfortunately these only indicate the location of the QRS complex, and not always the precise location of the R wave peak. On the other hand, the PTB data set does not include annotations with the location of QRS complexes, so these were first estimated using the HT detector. In order to obtain a ground-truth for both datasets, each recording was first upsampled to 10000 Hz, after which the *xcorr* algorithm was used to obtain precise locations. A similar procedure was previously used by Shaw and Savard (Shaw and Savard 1995) to obtain precise ground-truth locations in their data set.

#### Trigger jitter

To compare the trigger jitter obtained with the *slope* algorithm with the more computationally complex *xcorr* algorithm, both were tested on high-resolution ECG recordings from the PTB database, after downsampling^b^ them to different sampling rates (500, 200, 100, and 50 Hz). To illustrate that the localization precision is not only bound by the time resolution of the signal, but also by its bandwidth which is in turn limited by the sampling rate, both algorithms were also tested after upsampling each (downsampled) recording to the original sampling rate of 1000 Hz. Note that this procedure increases the time resolution but does not change the spectral content of the signal. For each algorithm and sampling rate, the pooled trigger jitter  was computed as
16

where *k* is the number of recordings in the data set and  is the standard deviation of the localization error (trigger jitter) for all *n*_*i*_ peaks localized within 40 ms of any ground-truth location^c^ for recording *i*.

#### Localization

In order to evaluate the robustness of the *slope* algorithm in the presence of artifacts and noise, the ECG signals in the MIT-BIH data set were degraded with clipping artifacts and Gaussian noise. Clipping artifacts were introduced to simulate saturation artifacts (Venkatachalam et al. 2011) and digital clipping. The following procedure was used:

- Remove baseline wandering using the filtering procedure described in an earlier section.
- Compute the median, *p*_*m*_, and the 99.9th percentile *p*_*M*_ of the amplitude of the ECG.
- Determine the clipping threshold *ct* for a given clipping factor *cf*17
- Clip the ECG signal,
18

In order to simulate a wide range of clipping and saturation artifacts, the following clipping factors were used: 1 (no clipping), 0.6, and 0.3. The last factor is meant as a lower bound for this type of artifacts, as it is unlikely that a segment of a recording where the ECG is distorted to that degree can still allow any useful analysis.

In addition to clipping, the localization precision was tested in the presence of Gaussian noise added to the ECG signal to obtain specific signal-to-noise-ratios (SNR): 20 dB, 10 dB, and 5 dB. For simplicity, the noise power in the original signal was considered negligible in comparison with the added noise.

The localization precision of the *slope* post-processing algorithm was evaluated with each of the three QRS detectors listed earlier on the ECG recordings of the MIT-BIH data set degraded with the conditions described above. For each recording, the mean and standard deviation of the localization error (distance between detected/localized peaks and the ground-truth locations) before and after post-processing were computed.

#### Template matching

The impact of localization on template matching was evaluated by comparing its effectiveness in rejecting false positive detections before and after the *slope* algorithm was used. Mock “false positive detections” were added to the list of base QRS detections, after which template matching was applied. Complexes with a correlation below certain thresholds were rejected, and the resulting positive predictive value (PPV) and sensitivity were computed. The following procedure was used:

- Use the HT detector to detect the base locations in each ECG recording of the MIT-BIH data set.
- Add a percentage *f*_*i*_ of randomly located false positives (‘mock false positives’) to the base list of locations. For example, a ‘mock false positive percentage’ *f*_*i*_ of 100% means that a number of mock false positives equal to the number of original locations will be added.
- Compute the median length *B*_*j*_ between consecutive localized QRS complexes for each ECG recording *j*.
- Compute a template as the signal average of all windows of length *B*_*j*_ for each recording *j*.
- Compute the correlation between the template of each recording and the ECG signal for each location on that recording.
- Determine the threshold *T* such that the sensitivity obtained after removing locations with a correlation lower than *T* remains above a specified limit. Compute the corresponding PPV (post_none_).
- Use the *slope* algorithm to obtain a list of adjusted locations.
- Compute a new template and the corresponding correlations with the adjusted locations.
- Determine the thresholds *T* such that the sensitivity obtained after removing locations with a correlation lower than *T* remains above a specified limit. Compute the corresponding PPV (post_slope_).

The choice of using a varying threshold instead of a fixed one serves the purpose of simultaneously evaluating the effect on the sensitivity and PPV of the algorithm. In turn, this allows an assessment of its performance for different scenarios where for example, sensitivity is more important than PPV, or vice-versa.

## Results

### Trigger jitter

**Figure 3:**
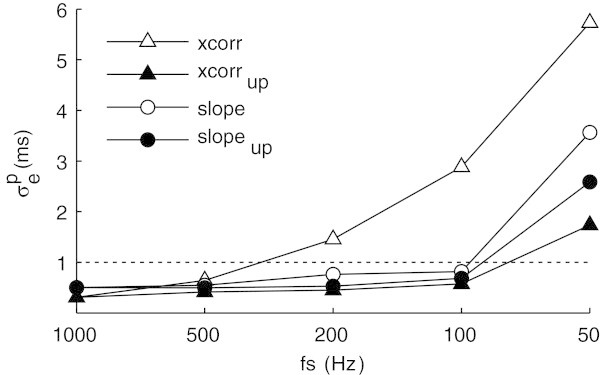
**Comparison of trigger jitter obtained using the** ***slope*** **algorithm with and without upsampling (‘slope’ and ‘slope**_**up**_ **’) and using the** ***xcorr*** **algorithm with and without upsampling (‘xcorr’ and ‘xcorr**_**up**_ **’).**

Figure 3 illustrates the pooled trigger jitter obtained with the *slope* and with the *xcorr* algorithms with and without upsampling, after having downsampled the original high-resolution ECG signal to different sampling rates. As it can be observed, the trigger jitter obtained with the *slope* algorithm for (down)sampling frequencies above 50 Hz was always lower than 1 ms, even without upsampling the signal, which means that the algorithm meets the strict requirement of 1 ms specified by the guidelines for QRS localization (Breithardt et al. 1991). In contrast, the trigger jitter obtained with the *xcorr* algorithm increased beyond 1 ms unless the signal was first upsampled. The localization error obtained with *xcorr* at 1000 Hz is not zero since the ground-truth was obtained after upsampling the original recordings at 10000 Hz.

The reason why the trigger jitter increased when the signal was downsampled to 50 Hz is related to the spectral properties of the QRS complexes. As reported by Thakor et al. (Thakor et al. 1984), QRS complexes have spectral components up to 40 Hz. When the signal is downsampled below 80 Hz, these components are affected, changing the morphology of the signal and causing a decrease in localization precision.

### Localization

**Figure 4:**
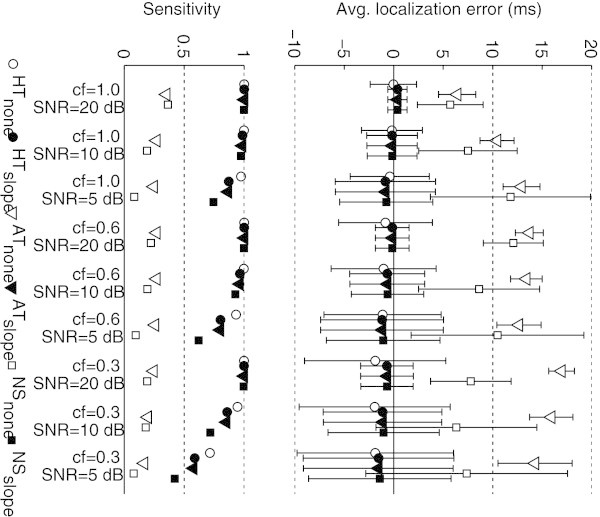
**Average localization error and trigger jitter for three QRS detection methods, before and after localization (subscripts ‘none’ and ‘slope’ respectively) for combinations of clipping factors and SNR (above), and corresponding sensitivity (below).**

The localization error and the trigger jitter were computed for all clipping artifact and noise conditions. The results, illustrated in Figure 4, show that the average localization error obtained after post-processing is, overall, lower than that obtained with the original locations output by the three QRS detectors, and always below 2 ms. In addition, the trigger jitter is also lower than 8 ms and lower than of the original locations with the exception of the AT detector. However, it should be noted that the sensitivity of this detector in these conditions was very low. After post-processing, sensitivity increased substantially, reflecting the increased precision in the localization of the complex.

**Figure 5:**
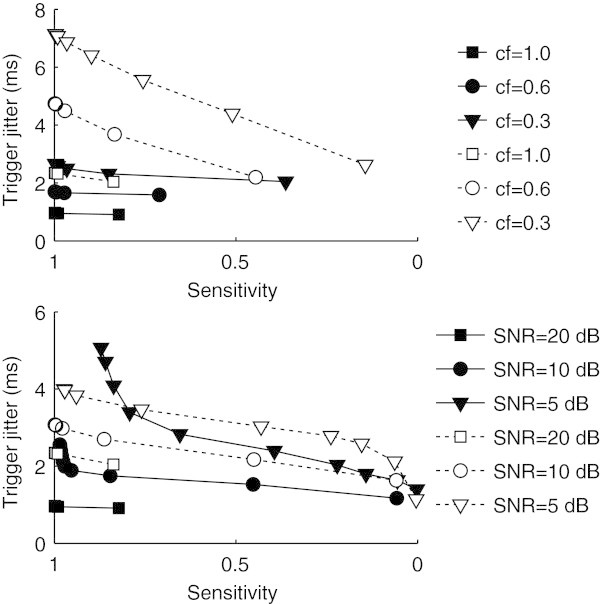
**Trigger jitter obtained after detection with HT, with and without localization (black-filled and white-filled markers, respectively) and template matching, for different clipping factors (above), and SNRs (below).**

To emphasize the improvement over the base HT performance, Figure 5 illustrates the trigger jitter obtained with and without *slope* localization after HT detection and after template matching. Varying the clipping factor for a fixed SNR of 20 dB (Figure 5, above) shows that the trigger jitter after localization (black-filled markers) is always lower than without localization (white-filled markers), highlighting the robustness of the algorithm to clipping artifacts. This is true for the whole range of sensitivities obtained using different thresholds with template matching. Varying the SNR without clipping (Figure 5, below) shows that with the exception of SNR = 5 dB, the trigger jitter is also always lower with *slope* localization, highlighting the robustness of the algorithm to noise, especially in moderate conditions. Regarding the condition SNR = 5 dB and if the analysis is restricted to complexes that are less distorted by noise (at the cost of a lower sensitivity), it is clear that below a sensitivity of 0.8 the trigger jitter with localization is also lower. Applications with strict requirements in terms of trigger jitter require this template matching step anyway to minimize the localization error. For such applications, the template matching procedure, with a complexity of *O*(*n*), does not increase the overall complexity of the *slope* algorithm.

These results are in line with the theoretical localization error derived in Appendix A.

### Template matching

**Figure 6:**
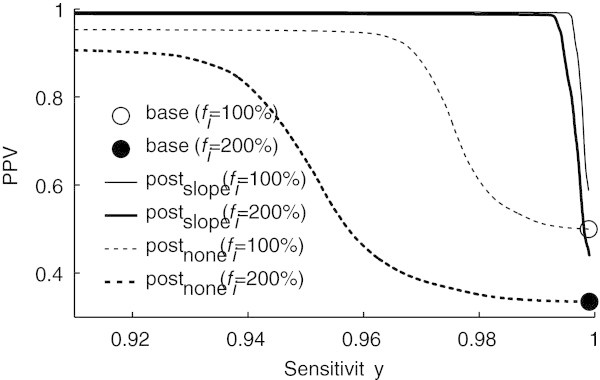
**Sensitivity vs. PPV after template matching using different correlation thresholds, with (‘slope’) and without (‘none’) localization for two false positive fractions** ***f***_***i***_ **.; indicated are also the base sensivity/PPV before localization and template matching; note that the x- and y-axes do not start at 0.**

Figure 6 illustrates the sensitivity and PPV obtained after rejecting locations with a correlation value below a varying threshold for mock false positive percentages, *f*_*i*_ = 100% and *f*_*i*_ = 200%. In both cases, increasing the threshold leads to the rejection of an increasing number of locations and to a consequent decrease in sensitivity, since besides false positives, a few true positives are also rejected. However, despite the fact that the sensitivity decrease is extremely small, the corresponding increase in PPV is substantial. Although this is true for both post_slope_ and post_none_, it is more pronounced for the case where post-processing is applied.

**Figure 7:**
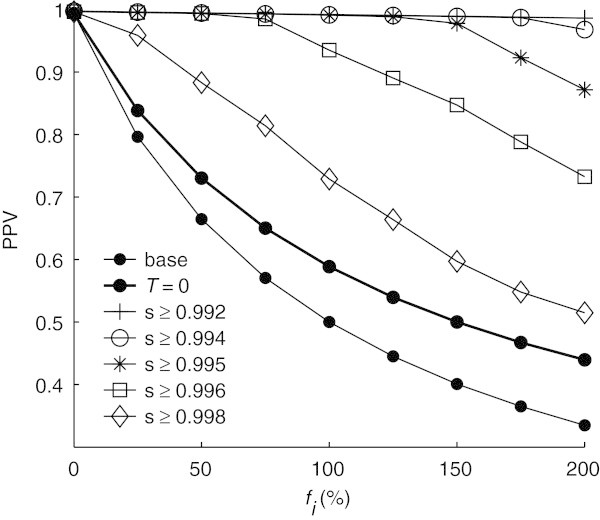
**PPV per false positive fraction** ***f***_***i***_ **after template matching with different thresholds; ‘base’ indicates the PPV without localization or template matching,** ***T*** **= 0 indicates the PPV after localization, without template matching, and each s ≥ X curve indicates the PPV after localization and template matching such that after rejecting locations with low correlation the resulting sensitivity is greater or equal than X.**

Figure 7 illustrates the PPV obtained for different *f*_*i*_ after rejecting peaks with correlations lower than thresholds chosen such that the sensitivity was higher than specific values. Note that even before locations are rejected, the PPV already increases after post-processing (*T* = 0). This happens because some false positive locations which are close to actual peaks in the ECG are merged to the same location on the first step of the localization algorithm (equation (2)). With a sensitivity of 0.992 the PPV approaches 1 even for . With a sensitivity decrease of 0.005 (0.5%) and 0.008 (0.8%) the PPV increases by nearly 50% and 70% for  and  respectively, effectively rejecting almost all false positives added to the base locations.

## Discussion and conclusions

Among the localization algorithms reported in literature, cross-correlation-based methods were found to be among the best performing. However, the localization precision of these algorithms is intrinsically linked to the sampling rate of the recording, which is not an issue for high-resolution ECG recordings, but is for recordings performed at lower sampling rates. In addition, these algorithms are computationally very expensive (*O*(*n*^2^), see Appendix B), which makes them less suited for low-power embedded devices; furthermore, since they typically require templates built from the entire recording, their applicability for real-time, online applications is limited. The algorithm proposed in this paper was developed with these requirements in mind. Despite its lower complexity (*O*(*n*)) it achieves comparable trigger jitter without requiring the data to first be upsampled. It also achieves high localization precision regardless of which QRS detector is used as input. Although it relies on the detection of just a few points in the ECG, it is extremely robust to noise, clipping and movement artifacts. In fact, post-processing with the *slope* algorithm was able to correct the original QRS locations of three different detectors under almost all noise conditions, yielding an average localization error below 2 ms, and a trigger jitter below 8 ms, even for recordings where the signal was severely degraded. Furthermore the increased localization precision improved template matching. After rejecting locations based on their (lower) correlation coefficient, it allowed the correct rejection of nearly all mock false positives added to a base list of detected QRS locations, with only a marginal decrease in sensitivity (0.5% for *f*_*i*_=100% and 0.8% for *f*_*i*_=200%).

These results have important consequences for applications which require real-time ECG analysis. Given its low complexity, low localization error and robustness to signal clipping, which is common in sleep recordings, it can enable real-time detection of arousals during sleep. On the other hand, given its robustness to noise and artifacts, it can also enable real-time ECG morphology analysis even under challenging conditions such as during physical exercise. Finally, and as a byproduct of the detection process, the slopes and tangents actually have clinical value, for example in detecting and characterizing myocardial ischemia (Pueyo et al. 2008; Romero et al. 2013).

## Endnotes

^a^Signal averaging is a technique used in QRS morphological analysis whereby a number of QRS complexes in an ECG recording are first aligned in the time domain and then averaged to improve the signal-to-noise ratio of the resulting signal.

^b^A low-pass filter with a cutoff frequency of 0.8*(fs/2), with fs as the desired frequency, was used before downsampling to prevent aliasing.

^c^This is the same margin as that indicated in the guidelines for analysis of ventricular late potentials using signal-averaging technique (Breithardt et al. 1991) and corresponds to half of the average length of QRS complexes (Rangayyan 2001).

^d^Using the slope estimates given by the localization algorithm, the minimum and maximum slope ratios found in the PTBD dataset were 0.68 and 1.84 respectively, confirming the expected asymmetry of QRS complexes described in literature (Pueyo et al. 2008; Romero et al. 2013).

## Appendix A – Theoretical localization error

This appendix derives the theoretical localization error obtained using the *slope* algorithm on an ECG signal contaminated with white Gaussian noise.

Consider the ascending and descending slopes of *y*_*A*_ and *y*_*D*_ as defined by (6) and (9) and assume that the ratio *β* between them is given by
1920

**Figure 8:**
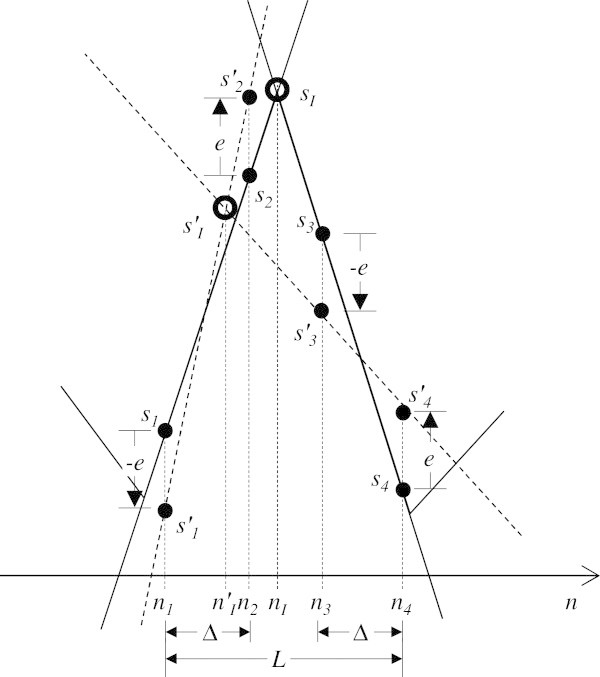
**QRS complex with tangents to the slopes of the signal (solid lines) and on noisy signal in the worst case scenario (dashed lines) yielding the maximum localization error for a noise component with an amplitude of 2** ***e.***

Now consider that the ECG signal has been perturbed by a noise signal with amplitude *E*. This means that in case both the QRS complex and the noise component have a zero mean, the amplitude of each sample in the QRS complex may change by a maximum of ± *e* = *E*/2. The worst possible localization error in this situation is illustrated in Figure 8.

The resulting tangents, *y'*_*A*_ and *y’*_*D*_, are defined by
2122

with
23242526

and intersect at a new location *n’*_*I*_,
27

Using (13) and (26), the maximum localization error, *e’*_*I*_ is given by
28

Noting that
29

we have that
30

Similarly,
31

Using the relation *n*_*4*_ *= n*_*1*_ *+ L* we have
32

Using (31) and (29) in (27), we finally have
33

As it can be seen, the localization error depends on the maximum amplitude of the noise component of the signal, the slopes of the QRS complex, the distance between the first and the last sample and the distance between each pair of samples (differentiation step) used to compute the tangents. For simplicity consider that the ascending slope *S* of the QRS complex is constant and defined by the ratio between the amplitude of the ECG signal A and half of the length of the QRS complex, *Q*/2. The localization error is then given by
34

Assuming that the noise component is mainly caused by electromyographic (EMG) noise, it can be considered to follow a zero-mean Gaussian distribution with a standard deviation of 10% of the amplitude of the ECG signal (Friesen et al. 1990). Taking as slope ratio *β* the median value experimentally found in the recordings of the PTBD dataset (1.13 ^d^), an average QRS duration of 0.08 sec (Rangayyan 2001), using a differentiation step Δ of 0.02 sec and choosing as *L* the length of the QRS complex Q (the largest possible value such that *n*_*1*_ and *n*_*4*_ are still part of the QRS), we can estimate the bounds of the standard deviation of the localization error (or trigger jitter),
35

## Appendix B – Computational Complexity

This appendix derives the computational complexity of the *xcorr* and *slope* algorithms and compares their performance using a computer simulation. Since for both algorithms peak localization takes place for every QRS complex, let *n* represent the average number of samples analyzed per complex. The *xcorr* algorithm performs the following steps:

1. Search for the local maximum, complexity *O*(*n*).
2. Add the contribution of each sample to the template (consider that the template has the same number of samples n), *O*(*n*) .
3. Perform cross-correlation with the template for 2*n* + 1 positions around the center of the peak, *O* (*n*⋅(2*n* + 1)) = *O*(*n*^2^) .
4. Find the position that yields the maximum cross-correlation, *O*(*n*) .
5. Repeat steps 2 to 4.

The overall complexity of the *xcorr* algorithm is *O*(*n*^2^). Note that the complexity of the upsampling step, necessary to guarantee a high localization performance with this algorithm for recordings with lower sampling rates was not included. Although that does not influence the overall complexity, it is an important factor to take into account when comparing the performance of both algorithms since it introduces an additional factor in the number of samples that need to be analyzed.

The *slope* algorithm performs the following steps:

1. Find the local maximum, *O*(*n*).
2. Differentiate the signal, *O*(*n*).
3. Find the maximum of the derivative before the local maximum, *O*(*n*/2) = *O*(*n*).
4. Find the minimum of the derivative after the local maximum, *O*(*n*/2) = *O*(*n*).

The overall complexity of the *slope* algorithm is *O*(*n*).

Both algorithms were also compared in terms of running time, using a non-optimized implementation in Matlab R2012b (The MathWorks Inc., Massachusetts) running on a computer with an Intel Core i5-2540 M at a clock speed of 2.60 GHz. Each algorithm was used to localize peaks detected with the Hamilton-Tompkins detector on the 18 recordings of the MIT-BIH data set. In the case of the *xcorr* algorithm, the signal was first upsampled to 1000 Hz to guarantee a comparable localization performance, but the running time for this operation was not included. The average running time (per recording) of the *xcorr* algorithm was 210.63 ± 55.31 sec and of the *slope* algorithm, 23.08 ± 6.26 sec, nearly ten times faster.

## References

[CR1] Afonso VX, Tompkins WJ, Nguyen TQ, Luo S (1999). ECG beat detection using filter banks. IEEE Trans Biomed Eng.

[CR2] Bangash MF, Xie A, Skatrud JB, Reichmuth KJ, Barczi SR, Morgan BJ (2008). Cerebrovascular response to arousal from NREM and REM sleep. Sleep.

[CR3] Bousseljot R, Kreiseler D, Schnabel A (1995). Nutzung der EKG-Signaldatenbank CARDIODAT der PTB über das Internet. Biomed Tech Eng.

[CR4] Bragge T, Tarvainen MP, Ranta-aho PO, Karjalainen PA (2005). High-resolution QRS fiducial point corrections in sparsely sampled ECG recordings. Physiol Meas.

[CR5] Breithardt G, Cain ME, El-Sherif N, Flowers NC, Hombach V, Janse M, Simson MB, Steinbeck G (1991). Standards for analysis of ventricular late potentials using high-resolution or signal-averaged electrocardiography. J Am Coll Cardiol.

[CR6] Catcheside PG, Chiong SC, Orr RS, Mercer J, Saunders NA, McEvoy RD (2001). Acute cardiovascular responses to arousal from non-REM sleep during normoxia and hypoxia. Sleep.

[CR7] EC57:1998/(R)2008 AS (1998). Testing and Reporting Performance Results of Cardiac Rhythm and ST Segment Measurement Algorithms.

[CR8] Ferreira JL, Cluitmans PJM, Aarts RM (2013). Detection of sharp wave activity in biological signals using differentiation between consecutive samples. 6th International Conference on Bio-inspired Systems and Signal Processing (BIOSIGNALS 2013).

[CR9] Friesen GM, Jannett TC, Jadallah MA, Yates SL, Quint SR, Nagle HT (1990). A comparison of the noise sensitivity of nine QRS detection algorithms. IEEE Trans Biomed Eng.

[CR10] Goldberger AL, Amaral LAN, Glass L, Hausdorff JM, Ivanov PC, Mark RG, Mietus JE, Moody GB, Peng CK, Stanley HE (2000). PhysioBank, PhysioToolkit, and PhysioNet: Components of a new research resource for complex physiologic signals. Circulation.

[CR11] Hamilton P (2002). Open source ECG analysis. Computers in Cardiology 2002.

[CR12] Hamilton PS, Tompkins WJ (1986). Quantitative investigation of QRS detection rules using the MIT/BIH arrhythmia database. IEEE Trans Biomed Eng.

[CR13] Jané R, Rix H, Caminal P, Laguna P, Jane R (1991). Alignment methods for averaging of high-resolution cardiac signals: A comparative study of performance. IEEE Trans Biomed Eng.

[CR14] Köhler BU, Hennig C, Orglmeister R (2002). The principles of software QRS detection. IEEE Eng Med Biol Mag.

[CR15] Moody GB, Mark RG (2001). The impact of the MIT-BIH arrhythmia database. IEEE Eng Med Biol Mag.

[CR16] Nygårds ME, Sörnmo L (1983). Delineation of the QRS complex using the envelope of the ECG. Med Biol Eng Comput.

[CR17] Pan J, Tompkins WJ (1985). A real-time QRS detection algorithm. IEEE Trans Biomed Eng.

[CR18] Pueyo E, Sornmo L, Laguna P (2008). QRS slopes for detection and characterization of myocardial ischemia. IEEE Trans Biomed Eng.

[CR19] Rangayyan RM (2001). Biomedical Signal Analysis: A Case-Study Approach.

[CR20] Redmond SJ, Heneghan C (2006). Cardiorespiratory-based sleep staging in subjects with obstructive sleep apnea. IEEE Trans Biomed Eng.

[CR21] Romero D, Ringborn M, Laguna P, Pueyo E (2013). Detection and quantification of acute myocardial ischemia by morphologic evaluation of QRS changes by an angle-based method. J Electrocardiol.

[CR22] Shaw GR, Savard P (1995). On the detection of QRS variations in the ECG. IEEE Trans Biomed Eng.

[CR23] Sörnmo L (1998). Vectorcardiographic loop alignment and morphologic beat-to-beat variability. IEEE Trans Biomed Eng.

[CR24] Stein PK, Pu Y (2012). Heart rate variability, sleep and sleep disorders. Sleep Med Rev.

[CR25] Task Force of the European Society of Cardiology and the North American Society of Pacing and Electrophysiology (1996). Heart rate variability. Standards of measurement, physiologic interpretation, and clinical use. Eur Heart J.

[CR26] Thakor NV, Webster JG, Tompkins WJ (1984). Estimation of QRS complex power spectra for design of a QRS filter. IEEE Trans Biomed Eng.

[CR27] Thayer JF, Yamamoto SS, Brosschot JF (2010). The relationship of autonomic imbalance, heart rate variability and cardiovascular disease risk factors. Int J Cardiol.

[CR28] Van Alsté JA, van Eck W, Herrmann OE (1986). ECG Baseline wander reduction using linear phase. Comput Biomed Res.

[CR29] Venkatachalam KL, Herbrandson JE, Asirvatham SJ (2011). Signals and signal processing for the electrophysiologist: part II: signal processing and artifact. Circ Arrhythm Electrophysiol.

[CR30] Volterrani M (1994). Decreased heart rate variability in patients with chronic obstructive pulmonary disease. Chest J.

